# Understanding the Impact of Cu-In-Ga-S Nanoparticles Compactness on Holes Transfer of Perovskite Solar Cells

**DOI:** 10.3390/nano9020286

**Published:** 2019-02-18

**Authors:** Dandan Zhao, Yinghui Wu, Bao Tu, Guichuan Xing, Haifeng Li, Zhubing He

**Affiliations:** 1 Department of Materials Science and Engineering, Shenzhen Key Laboratory of Full Spectral Solar Electricity Generation (FSSEG), Southern University of Science and Technology, No. 1088, Xueyuan Rd., Shenzhen 518055, Guangdong, China; yb77806@umac.mo (D.Z.); yinghui@cqu.edu.cn (Y.W.); 11653009@mail.sustc.edu.cn (B.T.); 2 Institute of Applied Physics and Materials Engineering, University of Macau, Macau SAR 999078, China; gcxing@umac.mo (G.X.); haifengli@umac.mo (H.L.)

**Keywords:** holes transport layer, compactness, hole transfer, recombination, Cu-In-Ga-S, perovskite solar cells

## Abstract

Although a compact holes-transport-layer (HTL) film has always been deemed mandatory for perovskite solar cells (PSCs), the impact their compactness on the device performance has rarely been studied in detail. In this work, based on a device structure of FTO/CIGS/perovskite/PCBM/ZrAcac/Ag, that effect was systematically investigated with respect to device performance along with photo-physics characterization tools. Depending on spin-coating speed, the grain size and coverage ratio of those CIGS films on FTO substrates can be tuned, and this can result in different hole transfer efficiencies at the anode interface. At a speed of 4000 r.p.m., the band level offset between the perovskite and CIGS modified FTO was reduced to a minimum of 0.02 eV, leading to the best device performance, with conversion efficiency of 15.16% and open-circuit voltage of 1.04 V, along with the suppression of hysteresis. We believe that the balance of grain size and coverage ratio of CIGS interlayers can be tuned to an optimal point in the competition between carrier transport and recombination at the interface based on the proposed mechanism. This paper definitely deepens our understanding of the hole transfer mechanism at the interface of PSC devices, and facilitates future design of high-performance devices.

## 1. Introduction 

The p-i-n inverted planar organic-inorganic perovskite solar cell (PSC), a device whose structural concept originates from the donor-acceptor structure of organic solar cells (OSCs) [1,2], was first developed by Guo et al. in 2013 [3], and has attracted great attention from researchers owing to its transporting balance of both holes and electrons, its simple device structure and its facile fabrication process, in contrast to the alternatives [4,5,6,7,8]. The initial planar heterojunction of perovskite/C_60_ is evolved into a core structure of perovskite/phenyl-C_61_-butyric acid methyl ester (PC_61_BM), which dominates almost all inverted planar PSCs structures, except for some inorganic components in place of PCBM [9,10,11]. At the same time, poly(3,4-ethylenedioxythiophene):polystyrene sulfonate (PEDOT:PSS) in the device structure acts as a good hole-transport layer (HTL) because it has the highest occupied molecular orbital (HOMO), which matches well with that of perovskites [12]. 

Although PEDOT:PSS has been used successfully as HTL material in inverted planar perovskite solar cells, it still has some problems: firstly, it does not remain stable at high temperatures; second, its intrinsically acidic properties can harm the perovskite layer. Thus, with regard to long-term stability, it is necessary to find a stable material as an alternative. In addition, inorganic P-type semiconductors are more chemically stable and have better carrier mobility, while also being cheap. To date, there have been some P-type inorganic materials applied in perovskite solar cells as the hole transport layer, including NiO [13,14,15], CuO [16,17], MoO_3_ [18], CuI [19,20], CuSCN [21,22,23,24], V_2_O_5_ [25,26,27], and so on. Among the inorganic P-type materials, Cu^+^–based oxychalcogenides, chalcogenides and halides are considered to be promising hole transport layers from the perspective of chemical modulation of the valance band. Thus, CZTS, CBTS, CIS, and so on have been used as the HTL in perovskite solar cells. CIGS, however, has usually been used as the bottom cell in tandem solar cells, with perovskite as the top cell, and few works have investigated CIGS as the hole transport layer in perovskite solar cells.

Here, we first applied CIGS nanoparticles as the HTL in inverted planar perovskite solar cells, which was obtained directly by spin coating the CIGS precursor solution onto the FTO substrate. As for CIGS, it has the advantages of chemical stability, bandgap tuning by means of composition engineering, and temperature and moisture stability in air. Therefore, it is promising to use CIGS to act as the HTL in perovskite solar cells in order to improve device stability.

It is well known that a dense, compact, and as thin as below 50 nm hole transport layer (HTL) film, as well as fully covering TCO (Transparent Conductive Oxides) electrodes, is important to block electron transport at the anode side and to keep low series resistance in devices [28]. Also, thickness and doping of HTLs were important extensively discussed to modulate the HTLs’ conductivity and reduce the band level offset in VBM (Valence Band Maximum) between perovskites and HTLs [29,30,31,32,33], while it is rarely investigated the compactness of HTL films really influences the extraction of holes and block of electrons [16,34,35,36]. In fact, the inverted planar PSCs with conversion efficiency as high as over 15% of were obtained in scarce of HTLs [37]. Cu_2_O nanoparticles was reported to modify ITO (Tin doped Indium Oxide) substrates to enhance the inverted planar PSC devices performance without HTL layers inside [36]. Therefore, the detailed investigation of what impact of the compactness of HTL films on the devices performance seems really compulsory.

In this work, a device structure of FTO (Fluorine doped Tin Oxide)/CIGS/perovskite/PC_61_BM/ZrAcac/Ag was adopted as the platform for this study based on the following two points. Firstly, so far, planar inverted PSCs on bare FTO substrates have had low conversion efficiency and severe hysteresis [38,39], thus affording space to investigate the effect of HTLs with a variety of coverages and thicknesses. Another advantage of FTO is its high temperature stability, which allows a broad process window. Secondly, CIGS is selected as the HTL in this work because it possesses such important merits as stable inorganic p-type materials, high hole mobility and concentration, direct and tunable bandgaps, high tolerance to defects and impurities, and long-term stability [40,41,42,43,44,45,46]. Although the bandgap of Cu-In-Ga-S is around 1.7 eV, the thickness of its HTL layer is too thin to absorb the incoming sunlight and to influence the light harvesting of the underlying perovskites. Because the Cu deficiency and vacancies can yield holes, the modulation of CIGS composition can tune hole concentration and further change its Fermi level and VBM. Along with tuning the bandgap, the conduction band minimum (CBM) of CIGS HTLs can also be regulated [42,47,48,49]. This enables the adaptability of CIGS to perovskites with small variation of VBM. In addition, the fabrication methods of CIGS films are so versatile and easy to scale up that they provide huge potential in terms of the commercialization of CIGS-based planar inverted PSCs [50,51,52,53,54,55]. Thanks to its room temperature solution process, CIGS film can be deposited by spin coating of precursor solution formed by facile dissolving of CuCl, InCl_3_, GaCl_3_, and thiourea in DMSO [40]. By changing the spin-coating speed, the coverage and thickness of CIGS films on FTO substrates can be tuned, leading to variation in device performance. By means of a series of photo physics tools, the working mechanism of the CIGS interlayer morphologies on device performance is explored theoretically, and a model is proposed to explain the competition between the carrier transport and recombination at the anode interface of the perovskite and the CIGS-modified FTO substrate. 

## 2. Experimental 

### 2.1. Materials

CuCl (99.999%), InCl_3_ (anhydrous, 99.99%) and PbI_2_ (99.9985%) were bought from Alfa Aesar (Beijing, China). Ga(NO_3_)_3_▪nH_2_O (99.99%), N, N-Dimethyl formamide (DMF), and Dimethyl sulfoxide (DMSO) were purchased from Sigma Aldrich (China). CH_3_NH_3_I was purchased from Taiwan Lumtec Corp. PC_61_BM (99.5%) was purchased from Shanghai MaterWin New Materials Co. (Shanghai, China), Ltd. All materials were used as received without further purification. 

Preparation of CIGS precursor solution: The CIGS solution was prepared according to the reported procedures with some modification. 0.0358 g Ga(NO_3_)_3_ ▪ XH_2_O was added after dissolving 0.2283 g thiourea, 0.0395 g CuCl, and 0.0729 g InCl_3_ in 4 mL DMSO, which were added consecutively to the ink and allowed to dissolve under stirring before adding the next. After the addition of Ga(NO_3_)_3_ ▪ nH_2_O, the inks were heated for 2 h at 120 °C to form a clear solution. All were prepared in the glove box (O_2_ < 50 ppm, H_2_O < 0.1 ppm).

### 2.2. Fabrication of CIGS Film

First, the FTO substrates were cleaned with DI water, toluene, acetone, and isopropanol for 20 min. Then, after spin coating the CIGS precursor solution on the FTO glass at different speeds (1000 r.p.m., 2000 r.p.m., 3000 r.p.m., 4000 r.p.m., 5000 r.p.m. and 6000 r.p.m.), the CIGS films were heated at 250 °C for 1–2 min on the preheated plate.

### 2.3. Device Fabrication 

First, the as-prepared perovskite precursor solution (PbI_2_ and CH_3_NH_3_I with mole ratio of 1:1 dissolved in a mixture of DMF and DMSO (2:1 v/v)) was filtered using 0.22 µm PTFE syringe filter and spin coated onto the CIGS films at a speed of 3500 r.p.m. for 35 s. During the last 15 s of the spinning process, the film was treated by gently drop-casting chlorobenzene solvent (200 µL). The substrate was then annealed on a hot plate at 100 °C for 10 min, followed by spin-coating a layer of PC_61_BM (2 wt. %, 1500 r.p.m.) on top and annealed for 20 min. Then, ZrAcac solution (1.0 mg/mL in methanol) was gently drop-casted (5000 r.p.m., 30 s) on top of PC_61_BM films. Finally, a 100-nm-thick Ag electrode was deposited through a shadow mask by thermal evaporation. The active area of the devices was 0.1 cm^2^.

### 2.4. Characterizations 

The J–V curves were recorded using a Keithley 2400 sourcemeter. Simulated solar illumination was provided by a Newport solar simulator under AM 1.5 at 100 mW cm^−2^ illumination with the start voltage at −0.2 V and the end voltage at 1.2 V, the delay time was 40 ms. The EQE spectra were recorded with by an Enli Technology (Taiwan) EQE measurement system (QE-R), and the light intensity at each wavelength was calibrated with a standard single-crystal Si photovoltaic cell. Absorption and transmission spectra were recorded by a UV/Vis/NIR Spectrophotometer (LAMBDA 950, PerkinElmer Waltham, MA, USA). Room temperature photoluminescence (PL) and time-resolved PL spectra were recorded by fluorescence spectrophotometer (FS5, Edinburgh Instruments Livingston, UK). A 405 nm pulsed laser was selected as excitation source for the time resolved PL measurement. Top-view scanning electron microscopy (SEM) images were characterized by MERLIN (Carl Zeiss AG, Jena, Germany) using an SE2 detector operating at an accelerating voltage of 5 KV. Cross-section scanning electron microscopy (SEM) was prepared using a focused ion beam (FEI Helios Nanolab 600i) operating at 30 KV and subsequently imaged with the electron beam of the same instrument using an accelerating voltage of 5 KV. The work function of FTO with or without CIGS films were measured by Ultraviolet photoelectron spectroscopy (UPS) (AXIS Ultra DLD, KRATOS Analytical Manchester, UK). The X-ray diffraction (XRD) patterns were recorded using a Bruker D8 X-ray diffractometer. An Atomic Force Microscope (AFM) (MFP-3D-Stand Alone, Asylum Research Abingdon-on-Thames, UK) was employed to investigate the film surface morphology.

## 3. Results and Discussion

**Figure 1:**
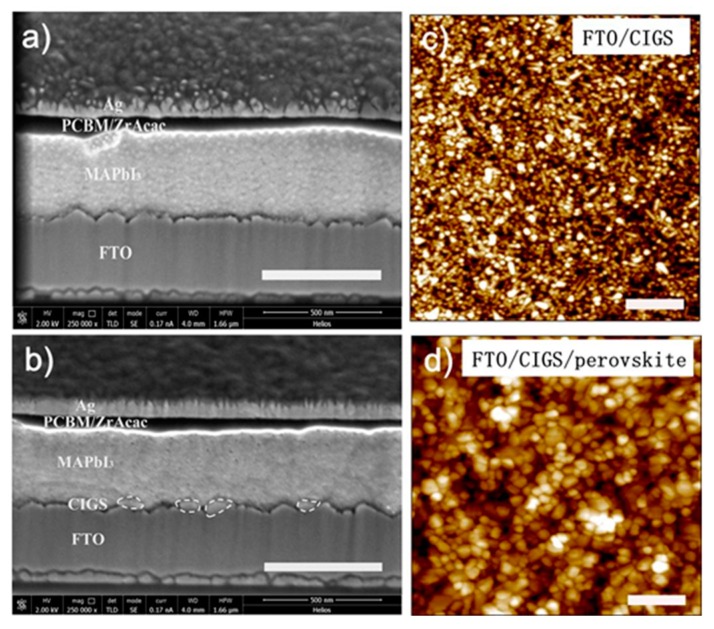
(**a**) Cross-section SEM image of the perovskite device without HTL layer. (**b**) Cross-section SEM image of the CIGS-based perovskite device. The scale bar is 500 nm. AFM images of the CIGS film (**c**) and the perovskite film on CIGS (**d**), respectively. The AFM image sizes are 5 μm × 5 μm and the scale bar is 1 μm.

Figure 1a,b shows cross-sectional SEM images of device structures of FTO/CIGS/perovskite/PCBM/ZrAcac/Ag without and with CIGS HTLs, respectively. The cross sections were prepared using the focus ion beam (FIB) tools. In both images, the MAPbI_3_ perovskite layer has an average film thickness of approximately 360 nm, which ensures the effective absorption of incident solar spectrum. At the top of the perovskite layer, a continuous layer of PCBM covers the entire perovskite surface with a thickness fluctuating from 30 to 90 nm, which is due to the roughness of the perovskite surface derived from FTO substrates. Then, a thin layer of ZrAcac was spin coated onto the surface of the PCBM to tune the working function of the silver electrodes that are approaching the conduction band level of the PCBM [56]. A continuous layer of PCBM/ZrAcac is highly important in preventing the cross immigration of silver atoms and iodine ions [57]. In contrast to the structure without the CIGS interfacial layer, the anode interface with the CIGS modification becomes rougher, as shown in Figure 1b. Meanwhile, it is hard to tell that a compact and dense CIGS thin film covers the surface of the FTO substrates in Figure 1b, which is deemed to be a basic requirement for HTLs. Regardless, the perovskite film coated on the CIGS film looks compact (Figure 1d). 

**Figure 2:**
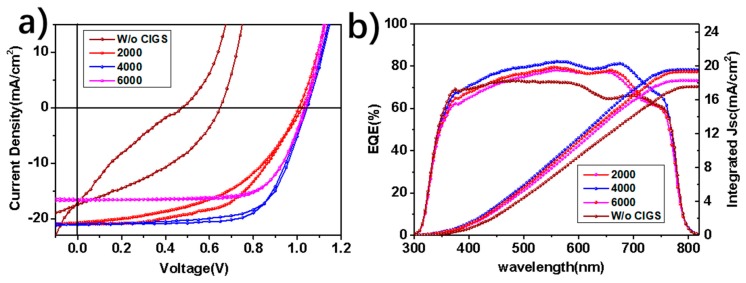
(**a**) ***J-V*** curves of PSCs with and without CIGS HTL layer, the active area of the devices is 0.1 cm^2^. (**b**) EQE spectra of the PSCs. The ***J_sc_*** were integrated from each EQE curve.

Thereafter, the device performances were investigated by tuning the coverage ratio and thickness of the CIGS films via spin coating speed (Figure 2). Without CIGS, the ***J-V*** curve shows severe hysteresis, and the device achieves a conversion efficiency of 4.3%, V_OC_ of 0.65 V, ***FF*** of 37.87% and ***J_SC_*** of 17.49 mA cm^−2^ in the backward scan. Without HTLs, the work function of FTO allows both holes and electrons to arrive at the perovskite/FTO interface without the blocking function of the electrons, leading to the intensive recombination of holes and electrons at the interface [38,39]. With the CIGS films, the open circuit voltages of the devices were improved remarkably to around 1 V, while also augmenting the fill factors (Figure 2a). In general, the higher the spin speed, the thinner the CIGS film. With a spin-coating speed of 6000 r.p.m., the ***V_OC_*** reaches 1 V, along with the suppression of hysteresis, which reveals that a very thin layer of CIGS can effectively decrease the interface recombination to a large extent. As its thickness increases with the successive reduction of the spin-coating speed from 6000 r.p.m. to 4000 r.p.m. and 2000 r.p.m., the ***V_OC_*** still has a small augmentation, while the ***J_SC_*** is enhanced remarkably, to close to 21 mA cm^−2^. Unexpectedly, the hysteresis becomes severe when the speed is decreased from 4000 to 2000 r.p.m., which demonstrates that some factors hinder the hole transfer efficiency and block the balance once again. This cannot be explained by the variation of CIGS thickness alone, and the relationship between the device performance and the speed dependent surface morphologies of CIGS interlayer is definitely nonlinear. Among all of the CIGS-modified devices, the most optimized device were recorded at the spin speed of 4000 r.p.m., and the best PCE was 15.16%, with a V_oc_ of 1.04 V, *J_sc_* of 20.93 mA cm^−2^, and *FF* of 69.77%. Figure 2b shows all of the EQE spectra, among which the device of 4000 r.p.m. shows the highest efficiency at over 80% in the visible light region. The integration of EQE spectra also confirms the current density tested by the simulator. The discrepancy between *J-V* and EQE is smaller than 5%, and belongs to the mismatch error [58].

**Figure 3:**
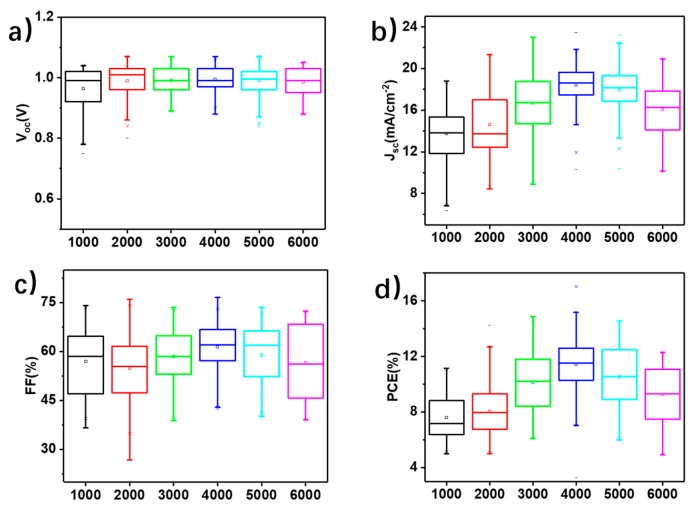
Comparison of the PSCs with CIGS thin films spin-coated at different speeds. Statistics for (**a**) V_oc_, (**b**) ***J_sc_***, (**c**) ***FF***, and (**d**) PCE.

In addition to the four representative kinds of devices, a series of devices with spin-coating speeds of the CIGS film ranging from 1000 r.p.m. to 6000 r.p.m. were investigated, and the results are summarized in Table S1, where the best values for each device parameter are listed in the forward and backward scans. For each kind of device, the performance data for a batch of 36 cells, measured in the same simulated solar spectra of AM 1.5G (100 mW cm^−2^), were collected and counted in the box chart in Figure 3. Compared to the device without CIGS, the average open circuit voltages of all the devices with different thicknesses of CIGS films were enhanced from around 0.5V to close to 1V, and show little difference, while they are obviously different with respect to *J_SC_* and *FF*. The large error bars for *J_SC_* and FF may be caused by the roughness of the FTO surface and the incomplete coverage of the CIGS nanoparticles. Therefore, it cannot be simply attributed to the thickness variation of the CIGS films. To figure out the mechanism behind this, the surface morphologies of all kinds of perovskite films just deposited on CIGS-coated FTO substrates were investigated (Figure S1). All the perovskite films show compact crystalline grains ranging in size from 100 nm to 600 nm. In addition, CIGS thin films with different spin speeds had little impact on perovskite crystal formation. In contrast to the bare FTO substrates (Figure S2b), the grain size of the perovskite film became smaller, clearly demonstrating the impact of the CIGS interfacial film. However, whatever the spin-coating speed of the deposition of CIGS, the variation in grain size and surface morphology of those perovskite films was really small. Hence, it is difficult to attribute the performance differences in the series of devices with CIGS to differences in their perovskite films.

**Figure 4:**
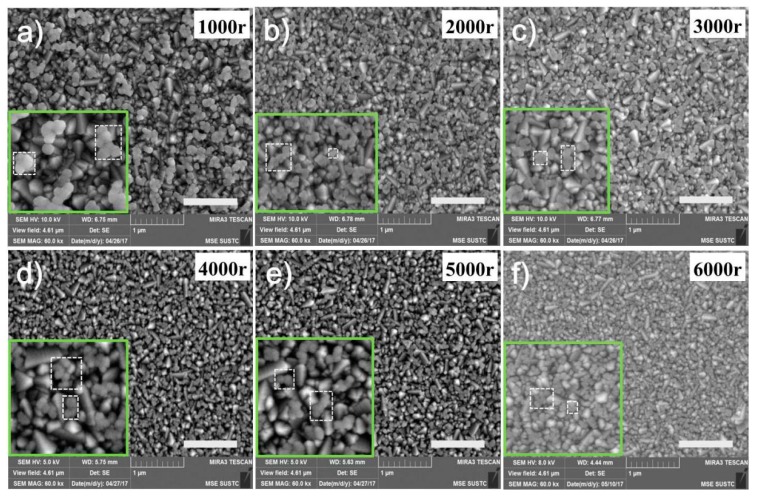
The morphologies of the CIGS thin film at different spin-coating rates on the FTO substrate. (**a**) 1000 r.p.m., (**b**) 2000 r.p.m., (**c**) 3000 r.p.m., (**d**) 4000 r.p.m., (**e**) 5000 r.p.m., (**f**) 6000 r.p.m. Scale bar is 1 µm.

Eliminating the influence of the quality discrepancy of perovskite films themselves, the CIGS interfacial layer itself becomes the critical issue that needs to be examined. According to a previous report [40], Cu_0.92_In_0.7_Ga_0.3_S_2_ thin films were fabricated by spin coating the CIGS molecular precursor solution onto sterilized FTO glass, which was formed by dissolving CuCl, InCl_3_, GaCl_3_, and thiourea in DMSO. Figure 4a–f shows the surface morphologies of the CIGS thin films spin coated at different spin-coating speeds ranging from 1000 r.p.m. to 6000 r.p.m., respectively. These films are composed of individual CIGS nanoparticles which only fill in the valleys of FTO substrates (Figure S2a), rather than fully covering the surface. As is generally known, the thickness of the thin film is related to the spin speed; that is, the higher the spin speed, the thinner the thin films and the smaller the nanoparticle size. The higher the spin speed, the smaller the nanoparticle size, but the higher the coverage ratio. Because the thickness of the CIGS will affect the transmittance, and the grain size will affect the roughness of the FTO surface, it will affect the performance of perovskite solar cells. In any case, the sharp corners of FTO grains were still exposed in all the samples. 

The composition of the CIGS nanoparticle films is characterized by Energy Dispersive Spectrum (EDS) mapping in Figure S3, where the elements Cu, In, Ga, and S are examined uniformly in CIGS nanoparticle film deposited on a bare glass substrate. The weak broad peak in the X-ray Diffraction (XRD) pattern of this CIGS nanoparticle film is indexed to the (112) plane of a CIGS chalcopyrite crystal structure (Figure S4), which also implies that the film is very thin and not high in crystallinity due to its low annealing temperature [42,51]. However, the low-temperature solution process of our strategy affords much broader range of applications for CIGS films. As Figure 4a–f shows, the nanoparticle size of each film shrinks, as shown in the magnified images in each figure, while the coverage ratio increases with the increase in spin-coating speed. With respect to the device performance comparison mentioned above, both the grain size and the coverage of the CIGS interlayer films determine the charge transfer at the interface of perovskite/CIGS. There must be an optimal point between grain size and coverage ratio, where it has the best charge transfer efficiency. The charge transfer efficiency would more or less decline. In addition, the results of the Hall measurement confirmed the P type of the CIGS nanoparticle films, with an excellent mobility of 10.4 cm^2^ V^−1^ S^−1^, and a carrier concentration of 8.57 × 10^14^ cm^−3^, which is critical for efficient hole transport. 

**Figure 5:**
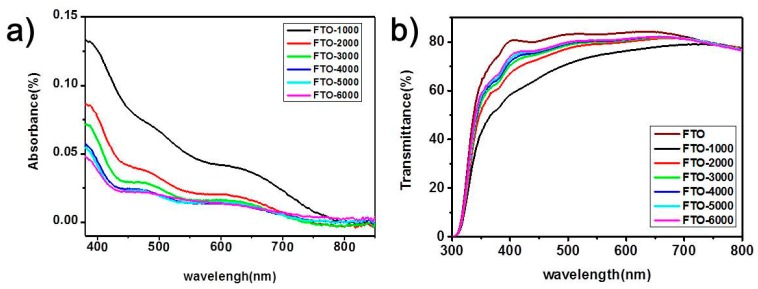
(**a**) Absorption spectra of the CIGS thin films at different spin-coating speeds. (**b**) Optical transmittance spectra of the pristine FTO and CIGS thin films at different spin-coating speeds.

On the other hand, the CIGS film in a FTO/CIGS/Perovskite/PCBM/ZrAcac/Ag device should be thin enough for light transmission, because the optical absorption edge of CIGS is around 740 nm. Figure 5a presents the absorption spectra of the series of CIGS thin films. The film spin-coated at 1000 r.p.m. exhibits an obvious absorption edge at around 740 nm, which is the typical one of CIGS films [59,60]. With the increase in spin speed, the nanoparticles become smaller and the films become thinner, and the CIGS absorptions becomes consequentially weaker, which eliminates our worry that this window layer would absorb the sunlight in advance of the underlying perovskite active layer. When the spin speed was higher than that at 1000 r.p.m., the optical band gaps of these films became larger with small variations. Figure 5b shows us a direct view of the transmission of these CIGS films. The transmission of these CIGS films decreases with the decrease in spin coating speed. This is because the thicker CIGS films derived from a lower spin speed allow less light to be transmitted due to both absorption and reflection by CIGS-modified FTO glass, leading to lower current density in the devices (Figure 2). However, the most optimized cell was recorded with 4000 r.p.m., rather than 5000 r.p.m. or 6000 r.p.m., which demonstrates that light transmission is not the only factor to determine the device current density and other performance parameters. Therefore, the band levels of our devices were investigated, as well as the hole transport ability of the CIGS interfacial layers, as described below.

**Figure 6:**
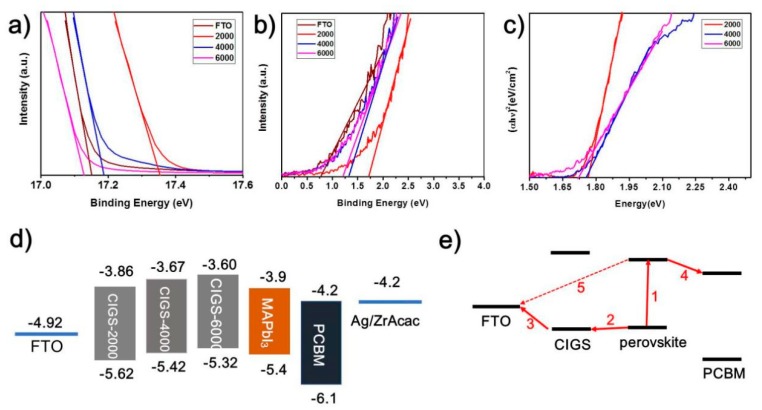
(**a**) and (**b**) are the UPS spectra of the CIGS thin films (spin coated at 2000 r.p.m., 4000 r.p.m., 6000 r.p.m., respectively) and FTO. (**c**) Tauc plots extracted from their corresponding absorption spectrum presented in Figure 3g. (**d**) Schematic diagram of charge transfer process in devices. (**e**) Band energy levels of each layer in the device as deduced from the data in Figure 4a–c.

Ultraviolet Photoelectron Spectroscopy (UPS) was applied to explore the band energy levels of these CIGS interfacial nanoparticles films (Figure 6). The work function was calculated by the equation E_F_ = 21.22 − E_cutoff_, and E_F_ is the Fermi level, and E_cuoff_ is the high binding energy cutoff. The work functions of CIGS-2000, CIGS-4000, and CIGS-6000 were 3.88 eV, 4.04 eV, and 4.10 eV, respectively. The valence band levels of the three typical kinds of CIGS-modified FTO substrates, along with bare FTO glass, were deduced from Figure 6a and 6b by the function E_VB_ = E_F_ + E_0,_ and E_VB_ is VBM, E_0_ is the low bounding energy tails [30,61]. The band gap of each corresponding kind can be calculated from Figure 6c. The band levels of the devices are collected in Figure 6d, which clearly depicts the difference in VBM mismatch between CIGS modified FTO substrates and perovskite films. The CIGS-4000 shows the best matching in VBM with that of the perovskite films, and the band level offset is the smallest (0.02 eV) among all the devices in this work. This accounts for the most efficient hole transfer at the anode interface. Meanwhile, the high CBM of each of the CIGS-modified FTOs also suppresses the electrons transferred from perovskite to FTO. This may be the main contribution to the augmentations of V_OC_ and *FF* for the CIGS-based devices with respect to the device on a bare FTO substrate [39]. 

Based on all the understandings mentioned above, Figure 6e shows plausible carriers transport routes in our devices. Firstly, electrons and holes depart from photon generated excitons (Step 1). Holes will go through CIGS nanoparticles (Step 2) and finally arrive at FTO electrodes (Step 3), while electrons will be extracted to PCBM (Step 4). Since the CIGS layer is not a continuous and dense film with FTO grain corners exposed, it is probable for electrons to reach the surface of FTO and recombine with the holes accumulated there (Step 5). Hence, a competition definitely exists between Step 5 and Step 3. Here, the grain size and coverage ratio of those CIGS interlayers would play a role in this competition, in addition to the band alignment effect of CIGS modifications. For CIGS-1000 or CIGS-2000, the grain sizes are larger, but the coverage is smaller. The hole transfer ability of CIGS nanoparticles will be limited by inadequate transfer sites, and hence their hole transfer efficiency is not high enough, and the recombination of carriers at this interface must occur substantially. As the spin-coating speed increases, the grain size shrinks, while the coverage ratio increases. When the grain size is below some critical value, as shown in CIGS-5000 and CIGS-6000, the charge transfer function of CIGS nanoparticles will deteriorate remarkably. Regarding the tradeoff between the grain size and coverage ratio of CIGS interlayers, CIGS-4000 seems to be the optimal with respect to this competition. Moreover, the variation trend of the hysteresis in J-V curves also implies that smaller grain sizes result in more efficient hole transfer.

**Figure 7:**
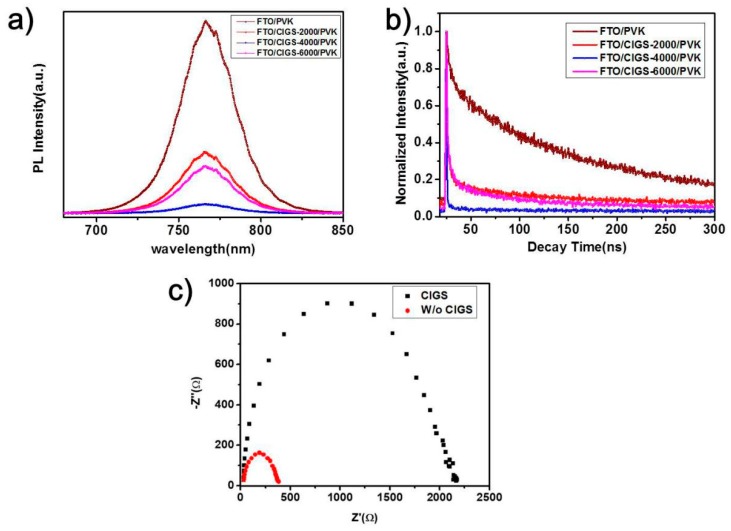
(**a**) Steady-state PL spectra and (**b**) time-resolved PL decays of perovskite films on the pristine FTO and the CIGS films at different spin speeds. (**c**) Nyquist plots for the PSCs with and without CIGS thin film as HTL.

To further confirm the working mechanism of CIGS interfacial layers, photoluminescence (PL) and time-resolved photoluminescence (TRPL) were used to characterize the perovskite films deposited on the four typical kinds of substrates. As displayed in Figure 7a, compared with the device without CIGS, the PL intensity was quenched significantly by the CIGS films, indicating effective hole extraction of the CIGS film. Comparing CIGS-2000, CIGS-4000 and CIGS-6000, CIGS 4000 shows the highest quenching rate. This phenomenon further confirms the mechanism illustrated in Figure 5. This coincides with the data of TRPL (Figure 7b). The PL decay curves were fitted using the biexponential decay function. The charge transfer time includes tow processes; namely, the fast decay process (τ_1_) and slow decay process (τ_2_). In addition, the fast decay represents the charge extraction from the perovskite to the CIGS film; the slow decay represents the radiative recombination in the perovskite [24,62,63]. The detailed data are summarized in Table S2. Among them, CIGS-4000 acts as the fastest hole extraction layer, with 0.69 ns of τ_1_, while that of the bare FTO, CIGS-2000, CIGS-6000 are 11.67 ns, 3.37 ns, and 2.92 ns, respectively. 

**Figure 8:**
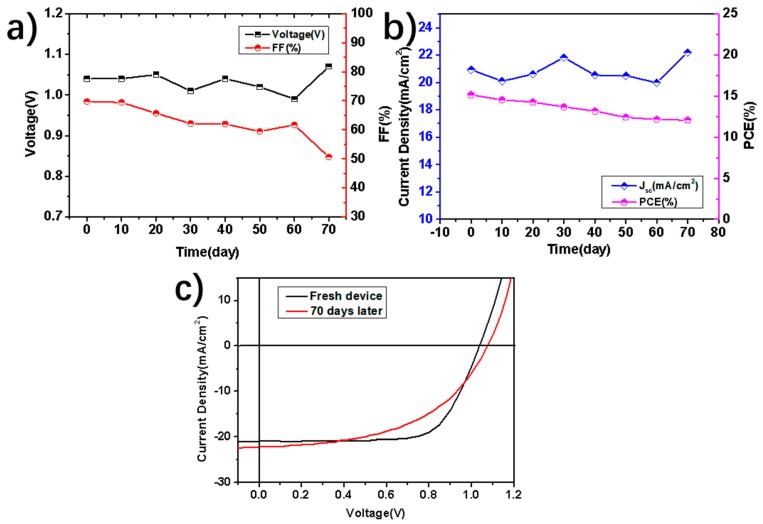
Long-term stability of the CIGS-based PSCs: (**a**) ***V_oc_*** and ***FF,*** and (**b**) ***J_sc_*** and PCE degradation of storage time in an inert atmosphere without encapsulation and tested in the air. (**c**) ***J-V*** curves of the fresh device and the same one after aging 70 days.

Figure 7c presents the Nyquist plot of the PSCs under dark at 0.7 V bias from 100 Hz to 1 MHz. As we know, the arcs at high frequency refer to the charge-transport resistance between the interface and perovskite, and those at low frequency are attributed to charge recombination [63,64,65]. Without CIGS modification, the smallest arc indicates rapid recombination of carriers at the interface between FTO and perovskite. Coinciding with the above results, with CIGS, the arc becomes larger, and therefore possesses slower carrier recombination, owing to the effective extraction by CIGS nanoparticles at the interface. Finally, the stability of the CIGS-4000 PSCs was checked. As for the chemical, oxygen and moisture stability in the air, the CIGS nanopartical layer can also protect the perovskite layer from being damaged. The device was tested at interval when stored in an inert environment without encapsulation. The parameters of J-V curves are shown in Figure 8a–b, and it retains almost 80% of its initial conversion efficiency value (Figure 8c). The FF degrades obviously, while the ***V_oc_*** and ***J_sc_*** are unexpectedly augmented. In any case, it exhibits good stability and great potential for future application.

## 4. Conclusions

In summary, the CIGS nanoparticles films were involved in evaluating the impact of the compactness of hole transport layers in the performance of PSCs. By changing the spin-coating speed from 1000 to 6000 r.p.m., the device at a spin-coating speed of 4000 r.p.m. achieved the optimal PCE of 15.16%, and V_oc_ of 1.04 V. In the UPS results, CIGS-4000 had the smallest band level offset (0.02 eV) with the perovskite, and achieved the most efficient hole transfer at the anode interface, also demonstrating that it was optimal in terms of the competition between recombination and transport at the interface. The optimum was yielded by a synergetic effect of both grain size and coverage ratio of the CIGS interlayer covering the surface of FTO electrodes. PL quenching and TRPL also confirm the variation law, along with the results of their Nyquist plots. This paper answers the question as to the necessary compactness of hole transport layers that is sufficient to separate holes from photon-generated carriers, and also definitely deepens the scope of our understanding of the detailed function of carrier transport layers.

## Supplementary Materials

The following are available online at http://www.mdpi.com/2079-4991/9/2/286/s1.

## References

[1] Etgar L., Gao P., Xue Z., Peng Q., Chandiran A.K., Liu B., Nazeeruddin M.K., Gratzel M. (2012). Mesoscopic CH_3_NH_3_PbI_3_/TiO_2_ heterojunction solar cells. J. Am. Chem. Soc..

[2] Meng L., You J., Guo T.-F., Yang Y. (2015). Recent advances in the inverted planar structure of perovskite solar cells. ACC. Chem. Res..

[3] Jeng J.Y., Chiang Y.F., Lee M.H., Peng S.R., Guo T.F., Chen P., Wen T.C. (2013). CH_3_NH_3_PbI_3_ perovskite/fullerene planar-heterojunction hybrid solar cells. Adv. Mater..

[4] Ball J.M., Lee M.M., Hey A., Snaith H.J. (2013). Low-temperature processed meso-superstructured to thin-film perovskite solar cells. Energy Environ. Sci..

[5] Liu M., Johnston M.B., Snaith H.J. (2013). Efficient planar heterojunction perovskite solar cells by vapour deposition. Nature.

[6] Jeon N.J., Noh J.H., Kim Y.C., Yang W.S., Ryu S., Seok S.I. (2014). Solvent engineering for high-performance inorganic-organic hybrid perovskite solar cells. Nat. Mater..

[7] Saliba M., Matsui T., Domanski K., Seo J.Y., Ummadisingu A., Zakeeruddin S.M., Correa-Baena J.P., Tress W.R., Abate A., Hagfeldt A. (2016). Incorporation of rubidium cations into perovskite solar cells improves photovoltaic performance. Science.

[8] Xing G., Mathews N., Sun S., Lim S.S., Lam Y.M., Grätzel M., Mhaisalkar S., Sum T.C. (2013). Long-range balanced electron-and hole-transport lengths in organic-inorganic CH_3_NH_3_PbI_3_. Science.

[9] Jung K.-H., Seo J.-Y., Lee S., Shin H., Park N.-G. (2017). Solution-processed SnO_2_ thin film for a hysteresis-free planar perovskite solar cell with a power conversion efficiency of 19.2%. J. Mater. Chem. A.

[10] Rao H.-S., Chen B.-X., Li W.-G., Xu Y.-F., Chen H.-Y., Kuang D.-B., Su C.-Y. (2015). Improving the Extraction of Photogenerated Electrons with SnO_2_ Nanocolloids for Efficient Planar Perovskite Solar Cells. Adv. Funct. Mater..

[11] Guo Q., Xu Y., Xiao B., Zhang B., Zhou E., Wang F., Bai Y., Hayat T., Alsaedi A., Tan Z. (2017). Effect of Energy Alignment. Electron Mobility, and Film Morphology of Perylene Diimide Based Polymers as Electron Transport Layer on the Performance of Perovskite Solar Cells. ACS Appl. Mater. Interfaces.

[12] Kim H., Lim K.-G., Lee T.-W. (2016). Planar heterojunction organometal halide perovskite solar cells: Roles of interfacial layers. Energy Environ. Sci..

[13] Wang K.-C., Jeng J.-Y., Shen P.-S., Chang Y.-C., Diau E.W.-G., Tsai C.-H., Chao T.-Y., Hsu H.-C., Lin P.-Y., Chen P. (2014). P-type mesoscopic nickel oxide/organometallic perovskite heterojunction solar cells. Sci. Rep..

[14] Zhu Z., Bai Y., Zhang T., Liu Z., Long X., Wei Z., Wang Z., Zhang L., Wang J., Yan F. (2014). High-Performance Hole-Extraction Layer of Sol–Gel-Processed NiO Nanocrystals for Inverted Planar Perovskite Solar Cells. Angew. Chem..

[15] Seo S., Park I.J., Kim M., Lee S., Bae C., Jung H.S., Park N.-G., Kim J.Y., Shin H. (2016). An ultra-thin. un-doped NiO hole transporting layer of highly efficient (16.4%) organic–inorganic hybrid perovskite solar cells. Nanoscale.

[16] Zuo C., Ding L. (2015). Solution-Processed Cu_2_O and CuO as Hole Transport Materials for Efficient Perovskite Solar Cells. Small.

[17] Chen L.-C., Chen C.-C., Liang K.-C., Chang S.H., Tseng Z.-L., Yeh S.-C., Chen C.-T., Wu W.-T., Wu C.-G. (2016). Nano-structured CuO-Cu_2_O complex thin film for application in CH_3_NH_3_PbI_3_ perovskite solar cells. Nanoscale Res. Lett..

[18] Tseng Z.-L., Chen L.-C., Chiang C.-H., Chang S.-H., Chen C.-C., Wu C.-G. (2016). Efficient inverted-type perovskite solar cells using UV-ozone treated MoOx and WOx as hole transporting layers. Sol. Energy.

[19] Christians J.A., Fung R.C., Kamat P.V. (2013). An inorganic hole conductor for organo-lead halide perovskite solar cells. Improved hole conductivity with copper iodide. J. Am. Chem. Soc..

[20] Qin P., Tanaka S., Ito S., Tetreault N., Manabe K., Nishino H., Nazeeruddin M.K., Grätzel M. (2014). Inorganic hole conductor-based lead halide perovskite solar cells with 12.4% conversion efficiency. Nat. Commun..

[21] Arora N., Dar M.I., Hinderhofer A., Pellet N., Schreiber F., Zakeeruddin S.M., Grätzel M. (2017). Perovskite solar cells with CuSCN hole extraction layers yield stabilized efficiencies greater than 20%. Science.

[22] Wijeyasinghe N., Regoutz A., Eisner F., Du T., Tsetseris L., Lin Y.H., Faber H., Pattanasattayavong P., Li J., Yan F. (2017). Copper (I) Thiocyanate (CuSCN) Hole-Transport Layers Processed from Aqueous Precursor Solutions and Their Application in Thin-Film Transistors and Highly Efficient Organic and Organometal Halide Perovskite Solar Cells. Adv. Funct. Mater..

[23] Ye S., Sun W., Li Y., Yan W., Peng H., Bian Z., Liu Z., Huang C. (2015). CuSCN-based inverted planar perovskite solar cell with an average PCE of 15.6%. Nano Lett..

[24] Jung J.W., Chueh C.C., Jen A.K.Y. (2015). High-Performance Semitransparent Perovskite Solar Cells with 10% Power Conversion Efficiency and 25% Average Visible Transmittance Based on Transparent CuSCN as the Hole-Transporting Material. Adv. Energy Mater..

[25] Liu Z., He T., Liu K., Zhi Q., Yuan M. (2017). Solution processed double-decked V_2_O_x_/PEDOT: PSS film serves as the hole transport layer of an inverted planar perovskite solar cell with high performance. RSC Adv..

[26] Liu Z., He T., Wang H., Song X., Liu H., Yang J., Liu K., Ma H. (2017). Improving the stability of the perovskite solar cells by V_2_O_5_ modified transport layer film. RSC Adv..

[27] Peng H., Sun W., Li Y., Ye S., Rao H., Yan W., Zhou H., Bian Z., Huang C. (2016). Solution processed inorganic V_2_O_x_ as interfacial function materials for inverted planar-heterojunction perovskite solar cells with enhanced efficiency. Nano Res..

[28] Mali S.S., Hong C.K. (2016). p-i-n/n-i-p type planar hybrid structure of highly efficient perovskite solar cells towards improved air stability: Synthetic strategies and the role of p-type hole transport layer (HTL) and n-type electron transport layer (ETL) metal oxides. Nanoscale.

[29] Liu M.H., Zhou Z.J., Zhang P.P., Tian Q.W., Zhou W.H., Kou D.X., Wu S.X. (2016). p-type Li. Cu-codoped NiOx hole-transporting layer for efficient planar perovskite solar cells. Opt. Express.

[30] Chen W., Liu F.-Z., Feng X.-Y., Djurišić A.B., Chan W.K., He Z.-B. (2017). Cesium Doped NiOx as an Efficient Hole Extraction Layer for Inverted Planar Perovskite Solar Cells. Adv. Energy Mater..

[31] Wei Y., Yao K., Wang X., Jiang Y., Liu X., Zhou N., Li F. (2018). Improving the efficiency and environmental stability of inverted planar perovskite solar cells via silver-doped nickel oxide hole-transporting layer. Appl. Surf. Sci..

[32] He Q., Yao K., Wang X., Xia X., Leng S., Li F. (2017). Room-Temperature and Solution-Processable Cu-Doped Nickel Oxide Nanoparticles for Efficient Hole-Transport Layers of Flexible Large-Area Perovskite Solar Cells. ACS Appl. Mater. Interfaces.

[33] Qiu Z., Gong H., Zheng G., Yuan S., Zhang H., Zhu X., Zhou H., Cao B. (2017). Enhanced physical properties of pulsed laser deposited NiO films via annealing and lithium doping for improving perovskite solar cell efficiency. J. Mater. Chem. C.

[34] Zhao K., Munir R., Yan B., Yang Y., Kim T., Amassian A. (2015). Solution-processed inorganic copper (I) thiocyanate (CuSCN) hole transporting layers for efficient p–i–n perovskite solar cells. J. Mater. Chem. A.

[35] Rao H., Sun W., Ye S., Yan W., Li Y., Peng H., Liu Z., Bian Z., Huang C. (2016). Solution-processed CuS NPs as an inorganic hole-selective contact material for inverted planar perovskite solar cells. ACS Appl. Mater. Interfaces.

[36] Liu L., Xi Q., Gao G., Yang W., Zhou H., Zhao Y., Wu C., Wang L., Xu J. (2016). Cu_2_O particles mediated growth of perovskite for high efficient hole-transporting-layer free solar cells in ambient conditions. Sol. Energy Mater. Sol. Cells.

[37] Li Y., Ye S., Sun W., Yan W., Li Y., Bian Z., Liu Z., Wang S., Huang C. (2015). Hole-conductor-free planar perovskite solar cells with 16.0% efficiency. J. Mater. Chem. A.

[38] Yin X., Que M., Xing Y., Que W. (2015). High efficiency hysteresis-less inverted planar heterojunction perovskite solar cells with a solution-derived NiOx hole contact layer. J. Mater. Chem. A.

[39] Chen W., Wu Y., Liu J., Qin C., Yang X., Islam A., Cheng Y.-B., Han L. (2015). Hybrid interfacial layer leads to solid performance improvement of inverted perovskite solar cells. Energy Environ. Sci..

[40] Uhl A.R., Katahara J.K., Hillhouse H.W. (2016). Molecular-ink route to 13.0% efficient low-bandgap CuIn(S,Se)_2_ and 14.7% efficient Cu(In,Ga)(S,Se)_2_ solar cells. Energy Environ. Sci..

[41] Todorov T.K., Gunawan O., Gokmen T., Mitzi D.B. (2013). Solution-processed Cu (In. Ga)(S, Se)_2_ absorber yielding a 15.2% efficient solar cell. Prog. Photovolt. Res. Appl..

[42] Mitzi D.B., Yuan M., Liu W., Kellock A.J., Chey S.J., Deline V., Schrott A.G. (2008). A High-Efficiency Solution-Deposited Thin-Film Photovoltaic Device. Adv. Mater..

[43] Garris R.L., Johnston S., Li J.V., Guthrey H.L., Ramanathan K., Mansfield L.M. (2018). Electrical characterization and comparison of CIGS solar cells made with different structures and fabrication techniques. Sol. Energy Mater. Sol. Cells.

[44] Jackson P., Hariskos D., Wuerz R., Kiowski O., Bauer A., Friedlmeier T.M., Powalla M. (2015). Properties of Cu (In. Ga) Se_2_ solar cells with new record efficiencies up to 21.7%. Phys. Status Solidi.

[45] Zhao D., Fan Q., Tian Q., Zhou Z., Meng Y., Kou D., Zhou W., Wu S. (2016). Eliminating fine-grained layers in Cu (In. Ga)(S, Se)_2_ thin films for solution-processed high efficiency solar cells. J. Mater. Chem. A.

[46] Xu L., Deng L.-L., Cao J., Wang X., Chen W.-Y., Jiang Z. (2017). Solution-Processed Cu (In. Ga)(S, Se)_2_ Nanocrystal as Inorganic Hole-Transporting Material for Efficient and Stable Perovskite Solar Cells. Nanoscale Res. Lett..

[47] Dullweber T., Rau U., Schock H. (2001). A new approach to high-efficiency solar cells by band gap grading in Cu (In. Ga) Se_2_ chalcopyrite semiconductors. Sol. Energy Mater. Sol. Cells.

[48] Gloeckler M., Sites J. (2005). Band-gap grading in Cu (In. Ga)Se_2_ solar cells. J. Phys. Chem. Solids.

[49] Zhang T., Yang Y., Liu D., Tse S.C., Cao W., Feng Z., Chen S., Qian L. (2016). High efficiency solution-processed thin-film Cu (In. Ga)(Se, S)_2_ solar cells. Energy Environ. Sci..

[50] Panthani M.G., Akhavan V., Goodfellow B., Schmidtke J.P., Dunn L., Dodabalapur A., Barbara P.F., Korgel B.A. (2008). Synthesis of CuInS_2_. CuInSe_2_, and Cu(InxGa_1−x_)Se_2_ (CIGS) nanocrystal “inks” for printable photovoltaics. J. Am. Chem. Soc..

[51] Zhao D., Tian Q., Zhou Z., Wang G., Meng Y., Kou D., Zhou W., Pan D., Wu S. (2015). Solution-deposited pure selenide CIGSe solar cells from elemental Cu. In, Ga, and Se. J. Mater. Chem. A.

[52] Wang G., Wang S., Cui Y., Pan D. (2012). A novel and versatile strategy to prepare metal–organic molecular precursor solutions and its application in Cu (In. Ga)(S, Se)_2_ solar cells. Chem. Mater..

[53] Jackson P., Hariskos D., Wuerz R., Wischmann W., Powalla M. (2014). Compositional investigation of potassium doped Cu (In. Ga) Se_2_ solar cells with efficiencies up to 20.8%. Phys. Status Solidi.

[54] Lin X., Klenk R., Wang L., Köhler T., Albert J., Fiechter S., Ennaoui A., Lux-Steiner M.C. (2016). 11.3% efficiency Cu (In. Ga)(S, Se)_2_ thin film solar cells via drop-on-demand inkjet printing. Energy Environ. Sci..

[55] Lee D.-Y., Park S., Kim J. (2011). Structural analysis of CIGS film prepared by chemical spray deposition. Curr. Appl. Phys..

[56] Chen W., Xu L., Feng X., Jie J., He Z. (2017). Metal Acetylacetonate Series in Interface Engineering for Full Low-Temperature-Processed. High-Performance, and Stable Planar Perovskite Solar Cells with Conversion Efficiency over 16% on 1 cm^2^ Scale. Adv. Mater..

[57] Chen W., Zhang G.-N., Xu L.-M., Gu R., Xu Z.-H., Wang H.-J., He Z.-B. (2016). Low temperature processed. high-performance and stable NiOx based inverted planar perovskite solar cells via a poly(2-ethyl-2-oxazoline) nanodots cathode electron-extraction layer. Mater. Today Energy.

[58] Cowan S.R., Wang J., Yi J., Lee Y.-J., Olson D.C., Hsu J.W. (2013). Intensity and wavelength dependence of bimolecular recombination in P_3_HT: PCBM solar cells: A white-light biased external quantum efficiency study. J. Appl. Phys..

[59] Park S.J., Cho J.W., Lee J.K., Shin K., Kim J.H., Min B.K. (2014). Solution processed high band-gap CuInGaS_2_ thin film for solar cell applications. Prog. Photovolt. Res. Appl..

[60] Li Q., Zhai L., Zou C., Huang X., Zhang L., Yang Y., Chen X.A., Huang S. (2013). Wurtzite CuInS_2_ and CuInxGa_1−x_S_2_ nanoribbons: Synthesis. optical and photoelectrical properties. Nanoscale.

[61] Chen W., Wu Y., Yue Y., Liu J., Zhang W., Yang X., Chen H., Bi E., Ashraful I., Grätzel M. (2015). Efficient and stable large-area perovskite solar cells with inorganic charge extraction layers. Science.

[62] Tan H., Jain A., Voznyy O., Lan X., de Arquer F.P.G., Fan J.Z., Quintero-Bermudez R., Yuan M., Zhang B., Zhao Y. (2017). Efficient and stable solution-processed planar perovskite solar cells via contact passivation. Science.

[63] Byranvand M.M., Kim T., Song S., Kang G., Ryu S.U., Park T. (2017). P-Type CuI Islands on TiO_2_ Electron Transport Layer for a Highly Efficient Planar-Perovskite Solar Cell with Negligible Hysteresis. Adv. Energy Mater..

[64] Lv M., Zhu J., Huang Y., Li Y., Shao Z., Xu Y., Dai S. (2015). Colloidal CuInS_2_ quantum dots as inorganic hole-transporting material in perovskite solar cells. ACS Appl. Mater. Interfaces.

[65] Wu Q., Xue C., Li Y., Zhou P., Liu W., Zhu J., Dai S., Zhu C., Yang S. (2015). Kesterite Cu_2_ZnSnS_4_ as a low-cost inorganic hole-transporting material for high-efficiency perovskite solar cells. ACS Appl. Mater. Interfaces.

