# Supplementary material for: Understanding the Impact of Cu-In-Ga-S Nanoparticles Compactness on Holes Transfer of Perovskite Solar Cells
Source: Nanomaterials (Basel). 2019 Feb 18;9(2):286. doi: 10.3390/nano9020286 (PMC6410191; doi:10.3390/nano9020286)
Supplement: Supplementary file 1 [file nanomaterials-09-00286-s001.pdf]

Supporting information

Understanding the Impact of Cu-In-Ga-S Nanoparticles Compactness on Holes Transfer of Perovskite Solar Cells

Dandan Zhao [1,2], Yinghui Wu [1], Bao Tu [1,2], Guichuan Xing [2], Haifeng Li [2] and Zhubing He [1,*]

[1] Department of Materials Science and Engineering, Shenzhen Key Laboratory of Full Spectral Solar Electricity Generation (FSSEG), Southern University of Science and Technology, No. 1088, Xueyuan Rd., Shenzhen, 518055, Guangdong, China; yb77806@umac.mo (D.Z.); yinghui@cqu.edu.cn (Y.W.); 11653009@mail.sustc.edu.cn (B.T.)

[2] Institute of Applied Physics and Materials Engineering, University of Macau, Macau SAR 999078, China; gcxing@umac.mo (G.X.); haifengli@umac.mo (H.L.)

* Correspondence: hezb@sustc.edu.cn; Tel.: +86-755-8801-8599

Table 1. Device parameters of the PSCs without and with CIGS nanoparticle interfacial layers coated on FTO substrates at different spin speeds ranging from 1000 rpm to 6000 rpm successively.

		V_{oc} V	J_{sc} mA cm^{-2}	*FF* %	PCE %
1000 r		0.96±0.06	13.7±2.50	56.93±9.88	7.58±1.53
	forward	1.01	15.22	72.34	11.15
	backward	1.02	15.26	70.14	10.89
2000 r		0.98±0.05	14.6±2.78	54.86±9.48	8.05±1.74
	forward	1.02	20.95	55.65	11.9
	backward	1.01	20.72	49.26	10.28
3000 r		0.99±0.04	16.7±3.01	58.37±8.19	10.11±2.18
	forward	1.04	21.53	63.89	14.24
	backward	1.05	21.88	64.59	14.88
4000 r		0.99±0.04	18.37±1.76	61.37±6.88	11.41±1.92
	forward	1.04	20.93	69.77	15.16
	backward	1.04	21.11	66.87	14.75
5000 r		0.98±0.05	17.92±2.09	58.87±8.89	10.52±2.23
	forward	1	20.05	70.55	14.21
	backward	1.01	20.22	68.44	14
6000 r		0.98±0.05	16.05±2.48	56.6±10.95	9.24±1.89
	forward	1.03	16.46	72.29	12.27
	backward	1.04	16.72	69.56	12.09
W/o CIGS		0.57±0.16	11.6±4.6	37.71±0.12	2.13±0.02
	forward	0.48	17.19	20.51	1.68
	backward	0.65	17.49	37.87	4.3

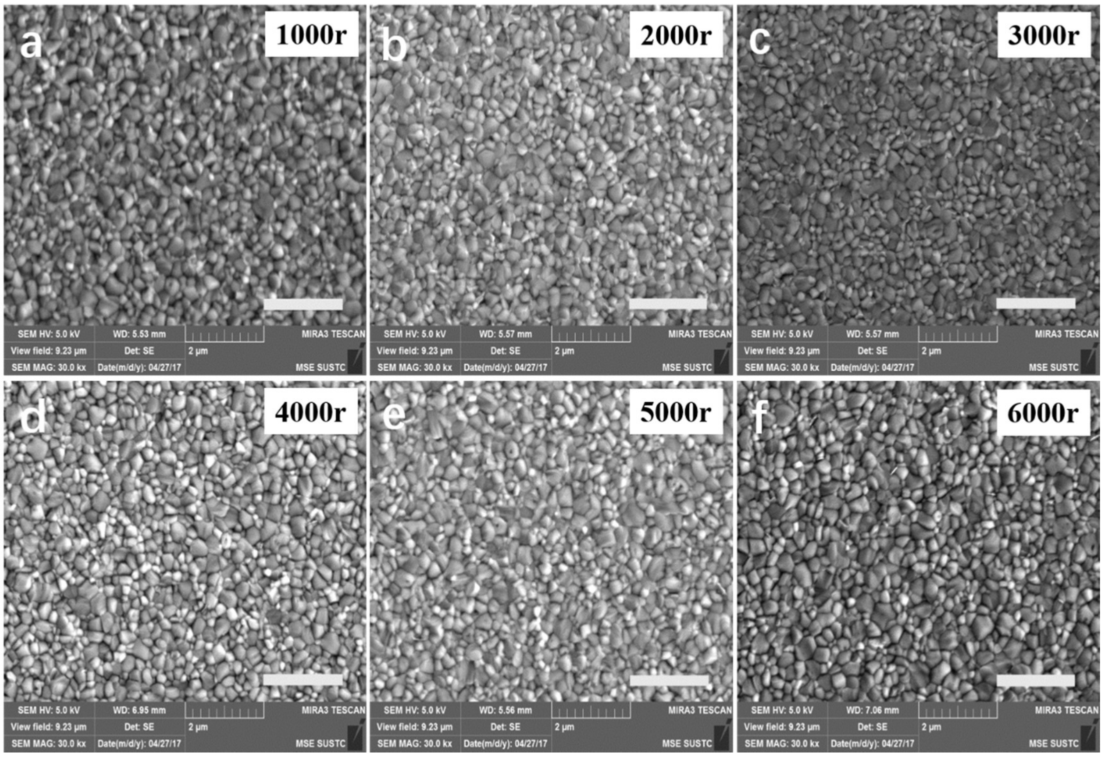

Figure S1. The SEM images of perovskite absorber layer on the CIGS nanopoarticle films of different spin speeds. (a) 1000 rpm, (b) 2000rpm, (c) 3000 rpm, (d) 4000 rpm, (e) 5000 rpm, (f) 6000 rpm.

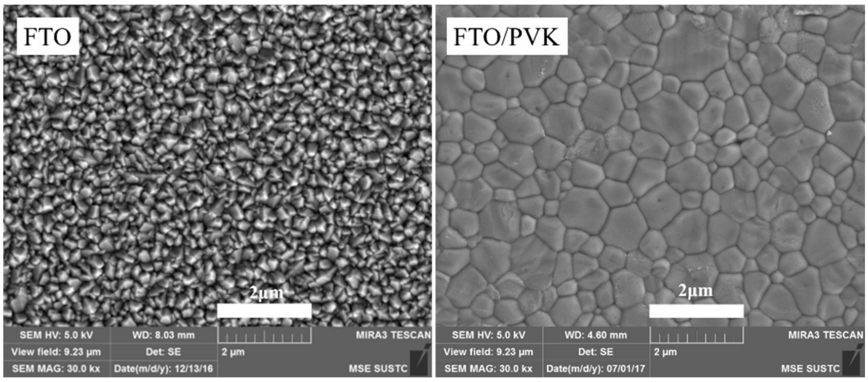

Figure S2. SEM images of the bare FTO (a) and the perovskite on bare FTO (b).

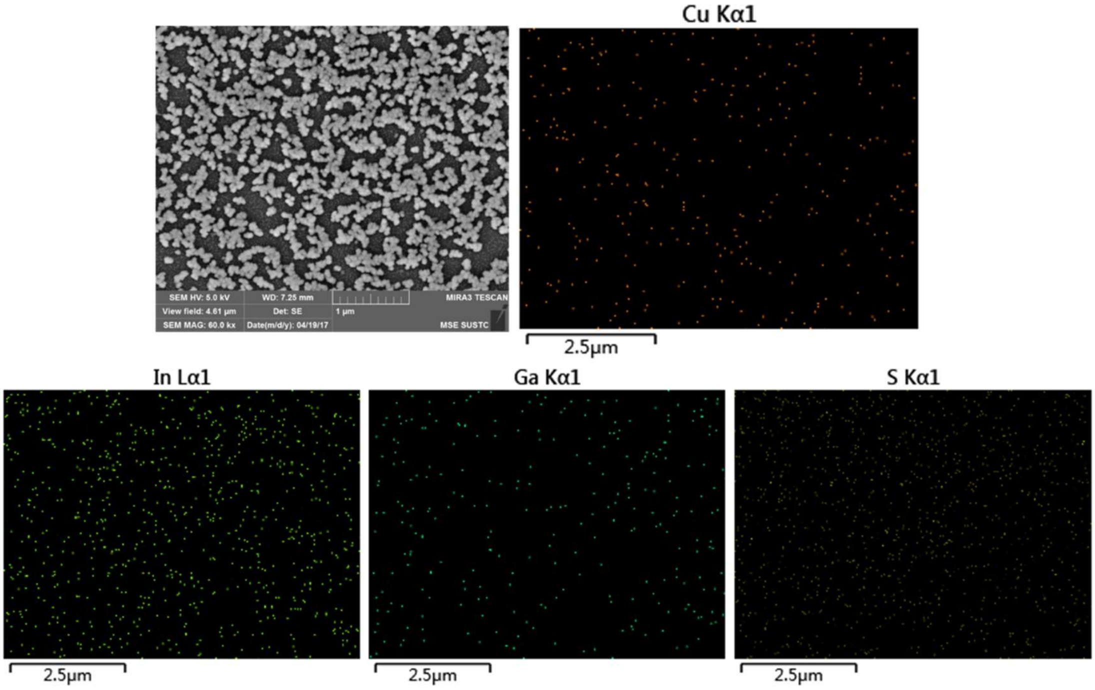

Figure S3. SEM image and EDS mappings of each element in the CIGS nanoparticle film on a bare glass substrate.

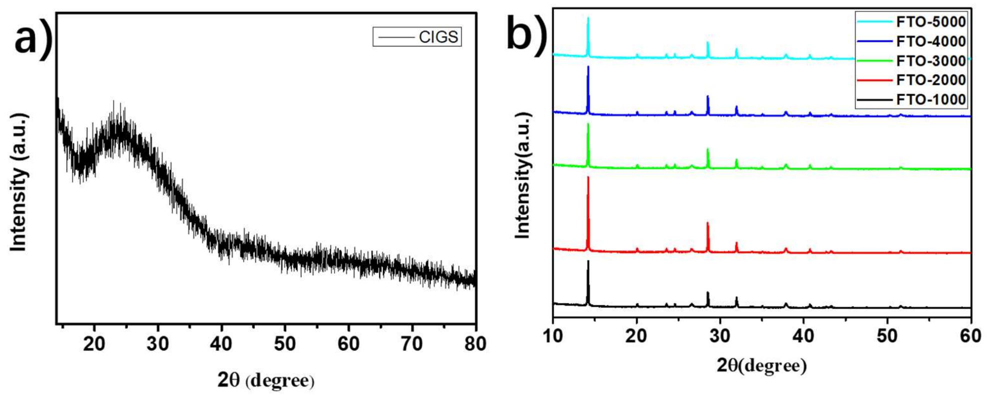

Figure S4. (a) XRD pattern of CIGS nanoparticle film annealed at 250 ^{o}C for 2 hrs, (b) XRD patterns of the perovskite films coated on those CIGS nanoparticle films of different spin speeds.

Table S2. Summary of the PL lifetime parameters of each kind of samples.

	A_1	τ_1	A_2	τ_2	τ_{ave}
FTO/PVK	462.20	11.67	1216.32	169.96	165.93
FTO/CIGS-2000/PVK	892.93	3.37	246.27	86.85	76.57
FTO/CIGS-4000/PVK	4362.88	0.69	30.59	120.67	66.59
FTO/CIGS-6000/PVK	1529.54	2.92	319.81	87.21	75.57
